# Chemoradiation After Pancreaticoduodenectomy for Pancreatic Adenocarcinoma is it of Proven Benefit?

**DOI:** 10.1155/1999/89794

**Published:** 1999-04

**Authors:** O. J. Garden

**Affiliations:** University Department of Surgery Royal Infirmary, Lauriston Place Edinburgh EH3 9YW UK


### Chemoradiation after Pancreaticoduodenectomy for Pancreatic Adenocarcinoma is it of Proven Benefit?

### ABSTRACT

*Yeo, C. J., Abrams, R. A., Grochow, L. B., Sohn, T. A., Ord, S. E., Hruban, R. H., Zahurak, M. L., Dooley, W. C., Coleman, J. A., Sauter, P. K., Pitt, H. A., Lilemoe, K. D. and Cameron, J. L. (1997) Pancreaticoduodenectomy for pancreatic adenocarcinoma: postoperative adjuvant chemoradiation improves survival. Annals of Surgery; 225, 621–636.*

**_Objective:_ This study was designed to evaluate prospectively survival after pancreaticoduodenectomy for pancreatic adenocarcinoma, comparing two different postoperative adjuvant chemoradiation protocols to those of no adjuvant therapy.**
**_Summary Background Data:_ Based on limited data from the Gastrointestinal Tumor Study Group. Adjuvant chemoradiation therapy has been recommended after pancreaticoduodenectomy for adenocarcinoma of the head, neck, or uncinate process of the pancreas. However, many patients continue to receive no such therapy.**
**_Methods:_ From October 1991 through September 1995, all patients with resected, pathologically confirmed adenocarcinoma of the head, neck, or uncinate process of the pancreas were reviewed by a multidisciplinary group (surgery, radiation oncology, medical oncology, and pathology) and were offered three options for postoperative treatment after pancreaticoduodenectomy: 1) standard therapy: external beam radiation therapy to the pancreatic bed (4000–4500 cGy) given with two 3-day fluorouracil (5-FU) courses and followed by weekly bolus 5-FU (500 mg/m$^2$ per day) for 4 months: 2) intensive therapy: external beam radiation therapy to the pancreatic bed (5040–5760 cGy) with prophylactic hepatic irradiation (2340–2700 cGy) given with and followed by infusional 5-FU (200 mg/m$^2$ per day) plus leucovorin (5 mg/m$^2$ per day) for 5 of 7 days for 4 months: or 3) no therapy: no postoperative radiation therapy or chemotherapy.**
**_Results:_ Pancreaticoduodenectomy was performed in 174 patients, with 1 in-hospital death (0.6%). Ninety-nine patients elected standard therepy, 21 elected intensive therapy and 53 patients declined therapy. The three groups were comparable with respect to race, gender, intraoperative blood loss, tumor differentiation, lymph node status, tumor diameter, and resection margin status. Univariate analyses indicated that tumor diameter <3 cm, intraoperative blood loss <700 mL, absence of intraoperative blood transfusions, and use of adjuvant chemoradiation therapy were associated with significantly longer survival ($p < 0.05$). By Cox proportional hazards survival analysis, the most powerful predictors of outcome were tumor diameter, intraoperative blood loss, status of resection margins, and use of postoperative adjuvant thereapy. The use of postoperative adjuvant chemoradiation therapy was a predictor of improved survival (median survival, 19.5 months compared to 13.5 months without therapy; $p = 0.003$). The intensive therapy group had no survival advantage when compared to that of the standard therapy group (median survival, 17.5 months *vs.* 21 months, $p$ = not significant).**
**_Conclusions:_ Adjuvant chemoradiation therapy signifcantly improves survival after pancreaticoduodenectomy for adenocarcinoma of the head, neck, or uncinate process of the pancreas. Based on these survival data, standard adjuvant chemoradiation therapy appears to be indicated for patients treated by pancreaticoduodenectomy for adenocarcinoma of the head, neck, or uncinate process of the pancreas. Intensive therapy conferred no survival advantage over standard therapy in this analysis.**

*Keywords:* Pancreatic adenocarcinoma, Pancreaticoduodenectomy, chemoradiation therapy

### PAPER DISCUSSION

Some ten years have elapsed since the Gastrointestinal Tumor Study Group recommended adjuvant chemoradiation therapy following pancreaticoduodenectomy for pancreatic adenocarcinoma [1,2]. There has been a reluctance, however, to accept the results of the study which demonstrated a small survival advantage for the treated patients. There was considerable scepticism regarding the significance of the findings of the study which recruited only 43 patients from a number of centres over an eight-year period. Whilst the results of other larger randomised studies are awaited, the surgeon has to look for guidance from institutions such as Johns Hopkins, where large numbers of patients have been managed with aggressive surgical and oncological protocols.

That surgery alone is insufficient to confer long-term survival benefit, is evident from this [3] and other recent papers from this group [4,5]. The median survival of the 174 patients undergoing pancreaticoduodenectomy was 19 months and actuarial survival was 36% at two years. The need to consider adjuvant treatment is emphasised by the fact that 51 (29%) of patients had positive resection margins and 130 (75%) had lymph nodes involved by tumour. The authors can justify their aggressive surgical approach based on a low complication rate (36%), a low re-operation rate (3%) and an in-hospital mortality of 0.6%. It is notable, however, that the 62 patients who developed complications had a median survival of 15 months as opposed to 20 months for the 112 patients who were complication-free. Other factors influencing survival included intra-operative blood loss, transfusion requirement and tumour diameter. In trying to establish whether chemoradiation after pancreaticoduodenectomy is of proven benefit, it is difficult to accept that these factors did not have a bearing on survival.

In the four-year study period, only patients who were deemed to have recovered satisfactorily from pancreaticoduodenectomy **by post-operative day 60** were encouraged to accept standard therapy or intensive therapy with chemoradiation. On this basis it is difficult to accept that the results of the two therapy groups can be usefully compared with the group of patients who did not receive post-operative chemoradiation. Median survival for the 53 patients who received no therapy was 13.5 months and compared unfavourably to the 21 months survival in the 99 patients who received conventional therapy comprising external beam radiation (EBR) to the pancreatic bed (4000–4500 cGy) and 5-Fluorouracil for four additional months. It was perhaps disappointing to find that the 21 patients who elected to receive more intensive EBR to the liver and pancreas, along with 5-Fluorouracil combined with leucovorin, did not appear to benefit, with a median survival of 17.5 months. It is a major concern of this non-randomised study that survival advantage was apparent within the first few months of follow-up, with approximately 95% of all patients receiving adjuvant therapy being alive at six months, compared to approximately 70% in the non-adjuvant group. Further analysis of the data shows that the no therapy group were significantly older, had a significantly longer post-operative stay in hospital and experienced significantly more complications than either of the two treatment groups. To be fair to the Baltimore group, their multivariate analysis did identify adjuvant therapy as a significant predictor of outcome. However, it could be argued that this conclusion was inevitable, given the patient and clinical bias towards selection of treatment. The lack of any quality of life assessment is an omission in a group of patients in whom the poor survival prospects render this a major consideration.

The results from this report may not readily lead other clinicians to agree with the authors' conclusions that standard 5-Fluorouracil-based chemotherapy combined with external beam radiation therapy appears to be indicated after surgery in patients undergoing pancreaticoduodenectomy for pancreatic adenocarcinoma. There is a need for more information and the results of randomised studies are eagerly awaited.

### *References*

[1] Kalser, M. H. and Ellenberg, L. S. S. (1985). Pancreatic cancer. Adjuvant conbined radiation and chemotherapy following curative resection. *Arch. Surg.*, **120**, 889–903.

[2] Gastrointestinal Tumor Study Group. (1987) Further evidence of effective adjuvant combined radiation and chemotherapy following curative resection of pancreatic cancer. *Cancer*, **59**, 2006–2010.

[3] Yeo, C. J., Abrams, R. A., Grochow, L. B. *et al.* (1997). Chemoradiation after pancreaticodenectomy for pancreatic adenocarcinoma: Is it of proven benefit? *Ann. Surg.*, **225**, 621–639.

[4] Yeo, C. J., Cameron, J. L., Lilemoe, K. D. *et al.* (1995). Pancreaticoduodenectomy for cancer of the head of the pancreas. 201 patients. *Ann. Surg.*, **221**, 721–733.
[5] Yeo, C. J., Cameron, J. L., Sohn, T. A. *et al.* (1997). Six hundred fifty consecutive pancreaticoduodenectomies in the 1990s: Pathology, complications, and outcomes. *Ann Surg.*, **226**, 248–260.

O. J. Garden, MD, FRCS (Glas & Ed)
Professor of Hepatobiliary Surgery
Director of Organ Transplantation
University Department of Surgery
Royal Infirmary, Lauriston Place
Edinburgh EH3 9YW United Kingdom

### Intractable Ascites Management: The Role of Side-to-Side Portacaval Shunt

### ABSTRACT

*Orloff, M. J., Orloff, M. S., Orloff, S. L. and Girard B. (1997) Experimental, clinical and metabolic results of side-to-side portacaval shunt for intractable cirrhotic ascites. The American College of Surgeons;* **184**, *557–570.*

***Background:*** **Intractable ascites, refractory to medical therapy, occurs in approximately 10 percent of patients with ascites from cirrhosis and is almost always fatal. Sinusoidal hypertension resulting from hepatic venous outflow obstruction plays a primary role in the pathogenesis of cirrhotic ascites and provides the rationale for decompression of the liver by side-to-side portacaval shunt in treatment of intractable ascites. This report presents the experimental basis for the use of side-to-side shunt and long-term results of a prospective study in 34 selected patients with intractable cirrhotic ascites.**
***Study Design:*** **In the experimental studies, hepatic venous outflow obstruction and massive ascites were produced in dogs by ligation of the hepatic veins, and the effect of portacaval shunts on ascites, thoracic duct lymph flow, and aldosterone secretion were measured. In the clinical study, 34 carefully selected patients with cirrhosis (91 percent alcoholic) and truly intractable ascites (failure of medical therapy for 5 to 24 months) underwent side-to-side portacaval shunt. The effects on ascites, survival, metabolic abnormalities, and quality of life were studied prospectively during follow-up that was longer than 5 years in all but two patients. Quantitative Child's risk classes in percent of patients were A in 0, B in 68 and C in 32.**
***Results:*** **In the experimental studies, side-to-side portacaval shunt permanently relieved severe ascites, reduced the 13-fold increase in thoracic duct lymph flow rate to almost normal, and abolished the aldosterone hypersecretory response to minimal hepatic venous outflow obstruction. End-to-side portacaval shunt was much less effective. In the clinical study, side-to-side portacaval shunt reduced mean portal vein-inferior vena cava pressure gradient from 282 mm saline to 4 mm and permanently relieved all patients of ascites without subsequent requirement of diuretic therapy. Two patients who died of hepatoma, and one who died of heart failure developed terminal ascites. Thirty-day mortality rate was 6 percent and long-term survival rates at 5, 10 and 15 years were 75 percent, 74 percent and 73 percent. In metabolic studies, side-to-side shunt produced marked diuresis and natriuresis and abolished hypersecretion of aldosterone. Quality of life was generally improved as a result of a low incidence of recurrent portal-systemic encephalopathy (6 percent), abstinence from alcohol in 91 percent, improvement in liver function in 81 percent, and improvement in Child's risk class. The portacaval anastomosis remained permanently patent in every patient.**
***Conclusions:*** **Side-to-side portacaval shunt is very effective treatment of intractable ascites from cirrhosis. Our results are attributable to careful selection of patients, an organized system of care, and a program of rigorous, lifelong follow-up that emphasizes abstinence from alcohol and dietary protein restriction. (J. Am. Coll. Surg., 1997;** ***184*****, 557-570).**

*Keywords:* Portacaval shunt, side-to-side shunt, ascites, refractory ascites

### PAPER DISCUSSION

The development of refractory ascites in cirrhotic patients is relatively low. It is accepted that approximately 10 percent of the patients with ascites either do not respond to diuretic therapy or develop diuretic-induced complications that

